# Preoperative treatment of locally advanced rectal cancer: less is more

**DOI:** 10.1002/mco2.443

**Published:** 2023-12-07

**Authors:** Shanshan Weng, Chenhan Zhong, Weijing Sun, Ying Yuan

**Affiliations:** ^1^ Department of Medical Oncology The Second Affiliated Hospital Zhejiang University School of Medicine Hangzhou China; ^2^ Cancer Institute, Key Laboratory of Cancer Prevention and Intervention Ministry of Education The Second Affiliated Hospital Zhejiang University School of Medicine Hangzhou China; ^3^ Division of Medical Oncology The University of Kansas Medical Center Kansas City Kansas USA; ^4^ Cancer Center Zhejiang University Hangzhou China

1

The PROSPECT study, a multicenter, unblinded, randomized, noninferiority trial, just released results in The New England Journal of Medicine. Schrag et al. have demonstrated that preoperative FOLFOX (contains 5‐Fluorouracil, Leucovorin and Oxaliplatin) regimen is non‐inferior to preoperative chemoradiotherapy (CRT) in terms of disease‐free survival (DFS) for patients with locally advanced rectal cancer (LARC) who are eligible for sphincter‐sparing surgery.^1^ This may therefore alter the preoperative treatment pattern of LARC: patients that meet its study inclusion criteria may be administrated neoadjuvant chemotherapy (nRT) alone.

In this study, patients with LARC that had been clinically staged as T2 node‐positive, T3 node‐negative, or T3 node‐positive and resectable were enrolled. Subsequently, they were randomized in a 1:1 ratio to either the FOLFOX group or the CRT group. Patients in the FOLFOX group received six cycles of modified FOLFOX6, while those who were unable to complete at least five cycles of FOLFOX or whose tumor had decreased in size by less than 20% were given CRT. Moreover, CRT is added after an efficacy assessment of six cycles of FOLFOX, and they still belong to the FOLFOX group. In both groups, the postoperative adjuvant chemotherapy of FOLFOX was suggested but not mandated. The primary endpoint of this study is DFS. A total of 1128 patients started treatment, 585 in the FOLFOX group and 543 in the CRT group. At a median follow‐up of 58 months, FOLFOX was non‐inferior to CRT for DFS (hazard ratio [HR] for disease recurrence or death, 0.92; 90.2% confidence interval [CI], 0.74–1.14; *p* = 0.005 for noninferiority). Five‐year DFS was 80.8% in FOLFOX group and 78.6% in the CRT group. The groups were similar with respect to overall survival (OS) and local recurrence.^1^ In this trial, neoadjuvant FOLFOX was an effective treatment option for patients who meet its inclusion with respect to local control, long‐term survival, and toxicity. Therefore, it can be concluded that these patients can forego radiotherapy. While the trial also has two main weaknesses. Firstly, the genetic status, such as mismatch repair (MMR), RAS, BRAF, and PI3KCa were not mentioned. Secondly, the trial did not include patients with T4 tumors, or sphincter‐preserving patients and other high‐risk factors (such as mesorectal fasciae [MRF] positive).

With the advancement of science and technology, researchers continue to explore the LARC treatment model (the shift of the treatment paradigm can be seen in Figure 1). The CAO/ARO/AIO‐94 study has demonstrated that neoadjuvant therapy for LARC can substantially improve the surgical resection rate and reduce local recurrence. As a result, the current standard therapy of LARC follows a “sandwich” approach, which consists of nCRT followed by total mesorectal excision (TME) and postoperative adjuvant chemotherapy. Although this mode can significantly improve the local control of rectal cancer and reduce the 5‐year recurrence rate to 5%–10%, the distant metastasis rate has not decreased significantly. Moreover, organ dysfunction caused by CRT+TME surgery and organ damage caused by combined abdominal perineal resection severely reduced the quality of life. Thus, researchers have raised many questions. How to optimize the treatment model? Is the potential of nRT underestimated? Then the notion of total neoadjuvant therapy (TNT), an alternative multimodal strategy meaning to intensify neoadjuvant treatment by delivering both radiotherapy and systemic chemotherapy, has been raised. The TNT approaches have shown benefits in the early prevention or eradication of micrometastases, higher rates of pathologic complete response (pCR) and longer DFS, and improving the tolerance and completion rates of chemotherapy. For some patients, surgery may be avoided if a complete response is achieved because of nRT. Based on this, the NCCN Panel recommends TNT as the preferred approach for high‐risk LARCs. Also, the results of the CONVERT study confirmed the efficacy of radiotherapy in LARCs. PROSPECT study is based on the TNT approach while including low‐risk LARC patients. The highlight of this study is some patients could forgo radiotherapy.

**FIGURE 1:**
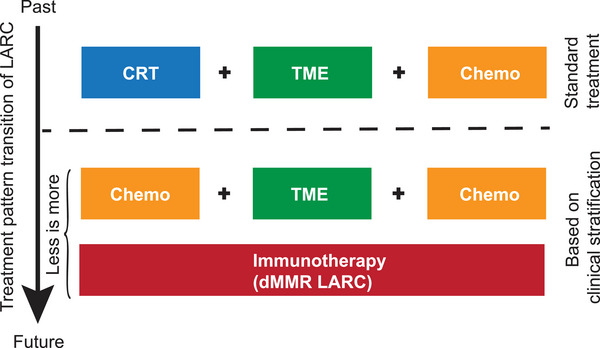
The treatment pattern transition of locally advanced rectal cancer (LARC). The current study demonstrated that preoperative 5‐Fluorouracil, Leucovorin, and Oxaliplatin (FOLFOX) are non‐inferior to preoperative chemoradiotherapy in terms of disease‐free survival for patients with LARC who are eligible for sphincter‐sparing surgery,^1^ which indicated that chemotherapy alone may be considered for some LARCs with low‐risk and moderate to high‐position tumors. In the future, the treatment pattern of LARC may be changed based on comprehensive consideration of clinical factors. Chemo: chemotherapy; CRT: chemoradiotherapy; TME: total mesorectal excision.

Can we modify the standard treatment approach without compromising the effectiveness of the treatment to suit the patient's needs? By omitting radiation, neoadjuvant systemic therapy could spare patients from radiation‐related morbidities. The phase III FOWARC trial compared neoadjuvant therapy with and without radiation and found that neoadjuvant FOLFOX without radiation resulted in lower pCR rates compared to regimens that included radiation (6.6%, 14.0%, 27.5% for mFOLFOX, fluorouracil‐RT, and mFOLFOX6‐RT groups, respectively). However, the FOWARC results did not demonstrate a significant improvement in DFS, local recurrence rates, or OS for FOLFOX with or without radiation compared to fluorouracil‐RT.^2^ When comparing the inclusion criteria of the FOWARC study with the PROSPECT study, the FOWARC study also recruited patients with T4, the patients who were of higher risk. Although the designs of these studies might differ, their results consistently suggested that nRT may potentially replace CRT in selected LARC patients (the inclusion of cited studies can be seen in Table S1). Thus, in some selected patients, nRT may be enough for them, which could preserve pelvic function while achieving good disease control.

Can we exclude chemotherapy and radiotherapy from neoadjuvant CRT (nCRT)? The results of the PSSR study indicated that initial surgery was inferior to conventional preoperative radiochemotherapy for locally advanced middle rectal cancer with magnetic resonance imaging (MRI)‐negative circumferential margins in terms of DFS.^3^ Therefore, reducing the treatment too much may adversely impact DFS. Also, it is not recommended to exclude chemotherapy and radiotherapy from nCRT.

Are there some patients with rectal cancer whose treatment methods can be simplified to the greatest extent and can achieve good therapeutic effects with only a single treatment method? In recent years, we have also witnessed significant advancements in immunotherapy and its application in deficient MMR (dMMR) patients. A prospective phase 2 study in which single‐agent dostarlimab, an anti‐PD–1 monoclonal antibody, was administered every 3 weeks for 6 months in patients with dMMR LARCs and showed pathologic responses in 100% of patients.^4^ Our overarching goal for dMMR patients is to streamline the treatment approach and achieve optimal therapeutic outcomes with a single treatment modality.

The findings in the PROSPECT trial have shown that neoadjuvant FOLFOX was an effective treatment option for selected patients. However, we have some concerns regarding its application in the clinical practice. Firstly, compared with other chemo‐only trials (excluding those trials consisting of bevacizumab), the pCR rate, namely 21.9% in the FOLFOX group of the PROSPECT study, is relatively high and may be owing to the inclusion of low‐risk patients and 9% of patients received CRT in the FOLFOX group. What kind of patients would be suitable for the treatment mode of adjuvant chemotherapy alone mentioned in this study? To date, accurate screening of patients is crucial. Based on previous results of RAPIDO^5^ and PRODIGE 23, those patients with a deeper degree of infiltration (cT4b and/or MRF positive) and low‐position tumors (< 5 cm from the anal verge) needed to be considered carefully due to their poor tumor regression. In addition, those patients who may have required abdominoperineal resection and equal or more than four lymph nodes with a short axis of 1 cm or more were excluded, and not all patients had pelvic MRI with disease assessment. With the well‐accepted TNT strategy in the clinic practice, the application of this study to the current practice needs carefully measured. Previously we have mentioned that neoadjuvant immunotherapy is suitable for dMMR LARC patients. Genetic status was not mentioned in this study, possibly because the study was initiated a decade ago when genetic status was not well understood. Thus, further studies are demanded, in which the situation of gene status should be considered. In addition, due to the development of sequencing technology, we have recognized the research advancement of liquid biopsy, which may also be helpful for risk stratification.

The results of the PROSPECT study showed that chemotherapy alone may be considered for some LARCs with low‐risk and moderate to high‐position tumors. However, the results should not be overestimated. In the future, more detailed clinical factors may be considered, such as risk classification, gene status, treatment conditions of the multi‐disciplinary treatment team, and patients' demands for function, to carefully select and treat patients with chemotherapy alone, thus bringing greater guiding significance to clinical practice.

## AUTHOR CONTRIBUTIONS

S.W. and C.Z. are co‐first authors and contributed equally to this work. S.W. and C.Z. conceived and drafted the manuscript. W.S. and Y.Y. provided valuable discussion and revised the manuscript. All authors have read and approved the final manuscript.

## CONFLICT OF INTEREST STATEMENT

The authors declare no conflict of interest.

## ETHICS STATEMENT

Not applicable.

## Supporting information

Supporting Information

## Data Availability

Not applicable.
