# Supplementary material for: Preoperative treatment of locally advanced rectal cancer: less is more
Source: MedComm (2020). 2023 Dec 7;4(6):e443. doi: 10.1002/mco2.443 (PMC10701462; doi:10.1002/mco2.443)
Supplement: Supplementary file 1 — Supporting Information [file MCO2-4-e443-s001.docx]

**Preoperative Treatment of Locally Advanced Rectal Cancer: Less is more.**

**Shanshan Weng^1,2#^, Chenhan Zhong^1,2#^, Weijing Sun^3*^, Ying Yuan^4*^**

1.Department of Medical Oncology, The Second Affiliated Hospital, Zhejiang University School of Medicine, Hangzhou, Zhejiang, China.

2.Cancer Institute, Key Laboratory of Cancer Prevention and Intervention, Ministry of Education, The Second Affiliated Hospital, Zhejiang University School of Medicine,

Hangzhou, Zhejiang, China.

3.Devision of Medical Oncology, The University of Kansas Medical Center, Kansas City, Kansas, United States.

4.Cancer Center, Zhejiang University, Hangzhou, Zhejiang, China.

^#^Shanshan Weng and Chenhan Zhong are co-first authors and contributed equally to this work.

**∗ Correspondence**

Ying Yuan, Department of Medical Oncology, The Second Affiliated Hospital, Zhejiang University School of Medicine, Hangzhou, Zhejiang, China.

Email: yuanying1999@zju.edu.cn

**Funding information**

Provincial Key R&D Program of Zhejiang Province, Grant/Award Number: 2021C03125; National Natural Science Foundation of China, Grant/Award Number: 8187248; Foundation from the Health Bureau of Zhejiang Province, Grant/Award Number: 2022489554.

| Table S1. The inclusion and brief results of cited trials | | |  |  |  |
| --- | --- | --- | --- | --- | --- |
| Study | n | Inclusion | Treatment | pCR（%） | Survival outcomes |
| PROSPECT(phase III) | 1194 | T2 node-positive, T3 node-negative, or T3 node-positive | neoadjuvant FOLFOX+/- radiotherapy; chemoradiotherapy | FOLFOX group: 21.9%, chemoradiotherapy group:24.3% | 5yr DFS: FOLFOX group: 80.8%, chemoradiotherapy group:78.6%. 5yr OS: FOLFOX group: 89.5%, chemoradiotherapy group:90.2%. |
| FOWARC(phase III) | 495 | stage II (T3-4N0) or stage III (T1-4N1-2), with a positive node defined as >= 1.0 cm in diameter on imaging) and a distal border located , 12 cm from the anal verge | fluorouracil-radiotherapy; mFOLFOX6-radiotherapy; mFOLFOX6 | fluorouracil-radiotherapy group: 14.0%, mFOLFOX6-radiotherapy: 27.5%; mFOLFOX6 groups:6.6% | 10yr OS:fluorouracil-radiotherapy group: 66.2%, mFOLFOX6-radiotherapy:73.2%; mFOLFOX6 groups:73%: |
| PSSR(phase III) | 299 | locally advanced rectal cancer (including all cT3/4 and/or Nany) with negative MRI-predicted CRM and tumor distance of 6 to 12 cm from anus | intervention group (surgery directly, in which positive CRM was supplemented with chemoradiotherapy, negative CRM received adjuvant chemotherapy according to surgical pathologic staging);control group (neoadjuvant chemoradiotherapy + surgery+ adjuvant chemotherapy). | control group:18.4%; intervention group: not available | 3yr DFS: intervention group 81.1%; control group 86.6% |
| Andrea Cercek(phase II) | 12 | mismatch repair–deficient stage II or stage III rectal cancer | dostarlimab only | cCR: 100% | At the time of this report, no patients had received chemoradiotherapy or undergone surgery, and no cases of progression or recurrence had been reported during follow-up (range, 6 to 25 months). |
| RAPIDO(phase III) | 920 | Locally advanced rectal adenocarcinoma, which was classified as high risk on pelvic MRI (with at least one of the following criteria: clinical tumour [cT] stage cT4a or cT4b, extramural vascular invasion, clinical nodal [cN] stage cN2, involved mesorectal fascia, or enlarged lateral lymph nodes), | experimental treatment group: short-course radiotherapy + CAPOX/FOLFOX4 + TME; standard of care group: radiotherapy + oral capecitabine + TME + CAPOX/FOLFOX4 | Experimental group: 28%;Standard of care group 14% | 3­yr OS: experimental group:89.1%; standard of care group:88.8% |
| Abbreviations: pCR, pathological complete responese; OS, overal survival; DFS, disease free survival | | | | |  |
